# Refinement of Structural Alerts for Hepatic Steatosis

**DOI:** 10.1021/acs.chemrestox.6c00156

**Published:** 2026-08-11

**Authors:** Anish Gomatam, James W. Firman, Georgios Chrysochoou, Larissa Camila Ribeiro de Souza, Steven J. Enoch, Judith C. Madden, Mark T. D. Cronin

**Affiliations:** School of Pharmacy and Biomolecular Sciences, 4589 Liverpool John Moores University, Byrom Street, Liverpool L3 3AF, U.K.

## Abstract

With the growing focus upon application of new approach methodologies (NAMs) within regulatory risk assessment of chemical substances, there is a clear need for human-relevant *in silico* models to predict toxicity associated with chemical exposure. This work describes the development of an *in silico* profiler for hepatic steatosis, a condition that results from the excess accumulation of fat within the liver. Toxicological data for chemicals were compiled from the literature, and expert judgments were made regarding their potential to induce steatosis. This list was used to test the 214 rules developed in a previous study. Many of these rules, originally derived from molecular initiating event (MIE)-based activation of ten nuclear receptor subtypes linked to liver injury, were found to be overly general, thereby limiting their predictive utility. These were supplemented with new fragments generated from the curated data set, which, following refinement, yielded 12 alerts indicative of steatosis. These alerts, 10 of which had precision ≥80%, captured chemicals with shared features and common toxicological profiles. Each was supported with a clear mechanistic rationale, by means of an extensive literature search, and, where possible, was linked to documented adverse outcome pathways (AOPs) describing steatosis. Ultimately, these were evaluated according to the established principles of uncertainty analysis and were shown to adhere to the findable, accessible, interoperable, and reusable (FAIR) Lite principles for data storage and sharing. The final collection of alerts has potential application in hazard identification, read-across, and screening/prioritization to support tiered assessment strategies targeting hepatic steatosis. To enable usage, a web-based implementation of the steatosis profiler that requires only chemical structures as input is available at https://steatosis-profiler.streamlit.app/.

## Graphical abstract

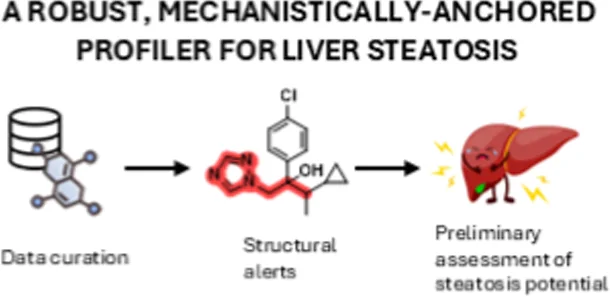

## 1 Introduction

Hepatic steatosis is a condition that results from increased lipid accumulation within the liver.^1^ Steatosis constitutes a major clinical concern and, if left untreated, can progress to advanced forms of liver disease.^2^ Although not itself a regulatory end point, its occurrence can serve as an important determinant in (sub)­chronic toxicity testing, potentially leading to lowered no observable (adverse) effect levels (NO­(A)­ELs)—particularly within conditions such as metabolic dysfunction-associated steatohepatitis and hepatitis C virus infections.^3,4^ It is widely acknowledged that, in addition to genetics and lifestyle-related factors, xenobiotic exposure may also trigger onset, with several studies linking the influence of environmental chemicals and pharmaceuticals to its progression.^5^

Preclinical assessment of steatogenic potential generally relies on animal models. However, for reasons relating to translatability, ethics, and cost, overt reliance upon these methods may prove problematic.^6^ As such, there has been a shift in the focus of toxicity testing toward new approach methodologies (NAMs), including *in chemico*, *in silico*, and *in vitro* techniques, which may be integrated into mechanism-based weight-of-evidence approaches for risk assessment.^7–11^ Thus, relevant *in silico* tools in support of tiered, mechanistically driven assessment strategies targeting this end point are highly desirable.

However, owing to the paucity of relevant data, *in silico* models focused upon steatosis are generally scarce. These have been limited to the development of quantitative structure–activity relationships (QSARs) or to machine learning-based classifiers.^12–14^ Gadaleta et al. used data from the ToxCast program to develop QSAR models characterizing several AOP-relevant molecular initiating events (MIEs) leading to steatosis, including upregulation of the pregnane X receptor (PXR), peroxisome proliferator activated receptor (PPAR), and the nuclear factor (erythroid-derived 2)-like 2 (Nrf2).^14^ Cotterill et al. assembled a data set of 207 compounds, classified as steatotic or nonsteatotic based upon *in vivo* histopathological findings,^12^ before constructing binary QSARs in the form of linear discriminant analyses. From a data set consisting of 1041 chemicals,^13^ Jain and co-workers constructed random forests relating to steatosis, which accounted also for nuclear receptor and transport protein perturbation.

While QSARs can provide useful estimates of toxicity potential, they often lack transparency.^15^ In contrast, structural alerts, defined as molecular fragments associated with specific forms of toxicity, can provide mechanistic insights in addition to flagging potential hazards.^16^ For example, within the adverse outcome pathway (AOP) framework, a structural alert can mechanistically support the link between chemical features, a plausible molecular initiating event (MIE), and an eventual adverse outcome.^17^

It is understood that as critical regulators of lipid accumulation and metabolism, nuclear receptors play a key role in the onset and progression of steatosis.^18,19^ This has led to the development of several AOPs linking modulation of key nuclear receptors to hepatic lipid and triglyceride accumulation, alongside an AOP network.^11^ These include AOP 36 (modulation of PPAR), AOP 57 (activation of the aryl hydrocarbon receptor), AOP 58 (suppression of the constitutive androstane receptor (CAR)), AOP 59 (activation of the PXR), AOP 61 (activation of the farnesoid X receptor (FXR)), and AOP 318 (activation of the glucocorticoid receptor).^20^ Against this background, Mellor et al. developed a set of 214 structural rules, based upon interaction at these sites, which, to the best of the authors’ knowledge, represents the only reported profiler focusing on steatosis as the apical end point.^21^

An appropriate assessment of the confidence that may be held within *in silico* models is crucial to their acceptance in the context of chemical safety assessment.^22^ With regard to structural alerts, this may be achieved through quantification of their associated uncertainties, alongside compliance with the findable, accessible, interoperable, and reusable (FAIR) principles of data sharing and storage.^23,24^ Cronin et al. described a “high, moderate, low” grading system as a practical means of assessing the confidence associated with structural alerts intended for toxicological end points.^23^ The scheme describes a total of 12 criteria, which encompass broadly the description of the alert, the consideration of mechanistic causality held with respect to adverse effects, the apparent level of performance exhibited, and the strength of supporting evidence. To assess computational toxicology models for compliance with the FAIR principles, Cronin et al. proposed the FAIR Lite scheme, which condenses the original FAIR framework into a practical checklist.^25^ Broadly, the FAIR Lite principles describe four criteria characteristic of open and accessible computational toxicology models: a unique identifier, model curation, metadata, and storage. As such, these schemas were deemed suitable for implementation within this study.

The objectives of this present investigation were to (a) curate a data set of chemicals, each judged and labeled in accordance with potential to induce steatosis, (b) develop a set of mechanistically driven structural alerts indicative of such potential, (c) assess the confidence associated with the alerts using a well-established scheme,^23^ and (d) disseminate a robust, validated *in silico* profiler in compliance with the FAIR Lite principles,^25^ intended to support risk assessment through hazard identification and read-across.

## 2 Methods

### 2.1 Collection and Curation of Steatosis Data

Data concerning the steatotic potential of chemicals were collected from six publications.^12,26–30^ The substances within consisted of pharmaceuticals, insecticides, pesticides, fungicides, simple organic molecules, and (organo)­metallic compounds. Each was assigned a binary activity label (steatotic/nonsteatotic) following an independent verification of the source data, e.g., regulatory sources such as European Food Safety Authority (EFSA) Cumulative Assessment Group (CAG) groupings documents or individual studies with a clearly described methodology, e.g., *in vivo* rodent or *in vitro* primary human hepatocyte studies. A brief of summary of the data sources is provided in Table 1.

**Table 1:** Literature Sources Used in the Compilation of the Steatosis-Annotated Data set of Chemicals^a^ ^b^

Author	Data Source	Description
Jain et al.^13^	RepDose, ToxRefDB, and HESS DB	RepDose: Database of experimental NOEL/LOEL values for various repeated dose toxicity end points^31^
		ToxRefDB: Rodent toxicology database (US Environmental Protection Agency)^32^
		HESS DB: Toxicity knowledge database with information on repeated dose toxicity and toxicity mechanisms^33^
Lichtenstein et al.^27^	EFSA CAG2b classification	Grouping of pesticides by EFSA into CAG2b, based upon *in vivo* phenomenological effects (hepatocellular fatty changes) observed in rodents^34^
Vrijenhoek et al.^28^	*In vivo* data drawn from multiple sources	Lipid accumulation or hypertrophy in preclinical repeated-exposure studies conducted as per OECD guidelines (e.g.: OECD Test Guideline No. 407: Repeated Dose 28 day Oral Toxicity Study in Rodents^35^), or following GLP
Kubickova et al.^29^	*In vitro* and *in vivo* data drawn from multiple sources	Consensus judgment based on review of multiple in vitro and *in vivo* studies
Escher et al.^30^	*In vitro* and *in vivo* data	Activity assigned based on GLP or OECD-compliant *in vivo* rodent studies, or selected MIEs and KEs within the AOP network for liver steatosis tested in human *in vitro* models
Cotterill et al.^12^	EFSA CAG2b groupings, ToxRefDB, and CEBS, ToxBank, and *in vivo* data from multiple studies	Pesticide data drawn from EFSA CAG2b groupings and rodent toxicology databases such as ToxRefDB and CEBS^37^ screened for chemicals with LEL < or = 100 mg/kg/day. Pharmaceutical data derived from multiple studies and the ToxBank data warehouse^38^

a CEBS: Chemical Effects in Biological Systems, GLP: Good Laboratory Practice, HESS DB: Hazard Evaluation Support System Database, KE: Key Event, OECD: Organisation of Economic Cooperation and Development, and ToxRefDb: Toxicity Reference Database.

b Data were integrated from six publications and were sourced from regulatory databases, European Food Safety Authority (EFSA) cumulative assessment group 2b (CAG2b) classifications, and independent *in vitro* and *in vivo* studies.

Data were compiled for a total of 1378 substances, the details of which are provided in Table S1 of the Supporting Information. Sufficient evidence through which to derive definitive judgments for association with steatosis could be obtained for 256 of this number, each of which was labeled as “high confidence”. This term was assigned to data meeting either of the following two criteria: (i) that which originated from regulatory sources, e.g., from EFSA CAG documents^34^ or from the US EPA’s Toxicity Reference Database (ToxRefDb);^32^ (ii) data from individual studies in which a clear description of the methodology was provided (e.g., Escher et al.)^30^ or where links to the source publications could be accessed and verified (e.g., Cotterill et al.).^12^ Entries that did not meet these criteria were not included in subsequent modeling efforts. The “high confidence” compounds were represented chemically as SMILES strings. Those corresponding to organometallics, inorganics, and larger biomolecules were identified and excluded using an in-house workflow built within KNIME software (Version 5.5.1).^39^ In its final form, the data set comprised 253 chemicals, of which 119 were positively associated with steatosis. A list of identifiers was assembled for each among these (presented within Table S2). All metadata associated with the development and validation of the alerts are available on the Zenodo repository (10.5281/zenodo.20236678).

### 2.2 Development of Structural Alerts

The 214 structural rules published in Mellor et al.,^21^ each relating to potential activation of nuclear receptors associated with steatosis were, alongside the 253 chemicals in the curated data set, investigated as potential sources of dedicated alerts. Substructure fragments were generated from the 253 compounds using the SARpy tool.^40^ Fragment size was set to a minimum of five and to a maximum of 18 atoms. These, together with the Mellor rules, were evaluated using an in-house KNIME workflow for their potential to correctly identify steatotic chemicals by screening them against the curated data set. This screening was carried out using the *Indigo Substructure Matcher* node available within the software. The following exclusion criteria were applied.

(i) Precision—The alerts were required to have a precision ≥0.6, in order to minimize false positives (calculated as shown in Equation 1).
(ii) Coverage—Fragments were required to have five occurrences or more, indicating that the substructure fragment occurs in >2% of the compounds.
(iii) Generality—Overly general alerts (e.g., linear aliphatic carbon chains) and ubiquitously occurring substructures (e.g., benzene rings) were discarded.

The performance of the 214 Mellor rules, alongside fragments generated from SARpy, was expressed in terms of number of true positives (TP) and false positives (FP) and general precision (as defined by Equation 1). All outcomes are presented within Tables S4A and S4B, respectively, of the Supporting Information.

1 $$precision=\frac{TP}{TP+FP}$$

where TP are steatosis-positive chemicals classified correctly and FP are steatosis-negative chemicals classified incorrectly. Precision is the ratio of TP to the total number of samples classified as positive (TP + FP).

The 214 Mellor rules, alongside the SARpy-generated fragments, were screened against the curated data set of chemicals using the same criteria, with failure to satisfy any single criterion resulting in exclusion. Fragments meeting these criteria were retained and refined using expert judgment, with the aim of improving coverage, regardless of their original source. To ensure an optimal balance between the generality and selectivity of coverage, features relating to atom type and aromaticity were either explicitly defined or kept general in scope, with restrictions placed on atomic connectivity wherever necessary. During this process, fragments originating from the Mellor rules and from SARpy were frequently combined into a single alert, and the nature of any refinement, i.e., retention, modification, or exclusion of structural features, was based upon whether the captured chemicals were already covered by another alert. Post refinement, both putative mechanisms of steatosis induction and relevant AOPs were assigned to the alerts by means of an extensive literature search.

### 2.3 Definition of the Applicability Domain

The applicability domain of the alerts was defined by using chemical descriptor ranges. Six physicochemical properties that describe features relevant to ligand–receptor binding were calculated, for each molecule within the data set, using the KNIME *Molecular Properties* node. These included an estimation of the logarithm of the octanol–water partition coefficient (XLogP), molecular weight, number of hydrogen bond acceptors and donors, topological polar surface area (TPSA), and number of rotatable bonds. Each is provided within Table S3A of the Supporting Information.

### 2.4 Assessment of Confidence

An assessment of the confidence associated with the refined alerts was carried out using the scheme proposed by Cronin et al.^23^ This describes 12 uncertainty criteria, relating broadly to the purpose, description, performance, and extent of evidence supporting each of the structural alerts.^23^ Expert judgment was applied to derive an overall level of confidence based upon their intended use cases.

### 2.5 Dissemination of a Robust *In Silico* Profiler in Compliance with FAIR Lite Principles

To ensure transparency and ease of accessibility, the alerts were assessed for compliance with the FAIR Lite principles outlined in Cronin et al.^25^ Briefly, these principles describe four aspects of models employed within the context of computational toxicology: (i) their accessibility by means of a unique identifier, (ii) the capture and curation of the model, (iii) the metadata for dependent and independent variables and, where possible, data, and (iv) storage in a searchable and interoperable platform.

### 2.6 Expansion of the Data Set to Improve Pharmaceutical Coverage

Following initial development of the profiler, the data set was further expanded to improve representation of pharmaceutical classes associated with steatosis. High-confidence data, supported by clinical case reports, regulatory drug labels, or the peer-reviewed literature, describing *in vivo* effects were recovered for 11 chemicals: two atypical antipsychotics, seven corticosteroids, and two nucleoside reverse transcriptase inhibitors (NRTIs). These chemicals, together with their respective sources, are presented in Table S2 of the Supporting Information. While the two NRTIs were subsequently found to be captured by an existing rule, it was necessary to construct two further alerts, corresponding to the antipsychotics and corticosteroids, following the same approach as previously described.

## 3 Results

### 3.1 Composition of the Data Set

Following curation, the final “high confidence” data set was composed of 253 substances, of which 119 were judged to be associated with steatosis. Details relating to each of these are provided within Table S2 of the Supporting Information. The data set was dominated by agrochemicals such as pesticides, fungicides, and insecticides, which collectively accounted for 172 entries. Also present were 45 pharmaceuticals, alongside 36 compounds not classified elsewhere, such as solvents, plasticizers, and industrial chemicals (grouped together under the ‘miscellaneous’ category). The distribution of these, by category and steatosis labels, is shown in Figure 1. These were supplemented with 11 additional chemicals covering previously underrepresented pharmaceutical classes, as described in Section 2.6.

**Figure 1:**
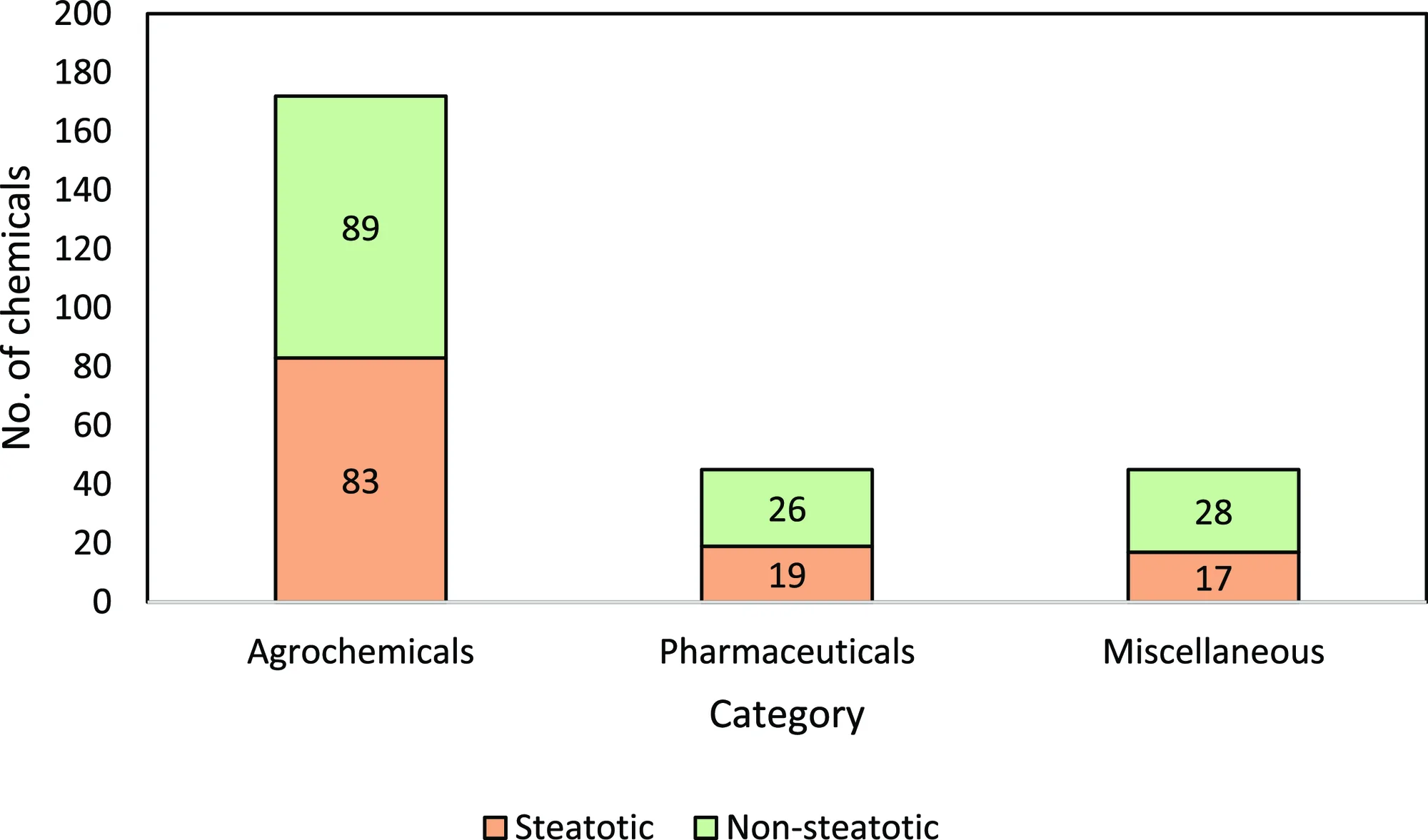
Number of chemicals, grouped by use category and steatosis labels. The data set has a relatively even distribution of steatotic vs nonsteatotic compounds. 11 additional chemicals were curated at a later stage (not shown here).

### 3.2 Development of Structural Alerts

A schematic depiction of the workflow, outlining the steps within data collection, alert development, and subsequent assessment, may be found in Figure 2.

**Figure 2:**
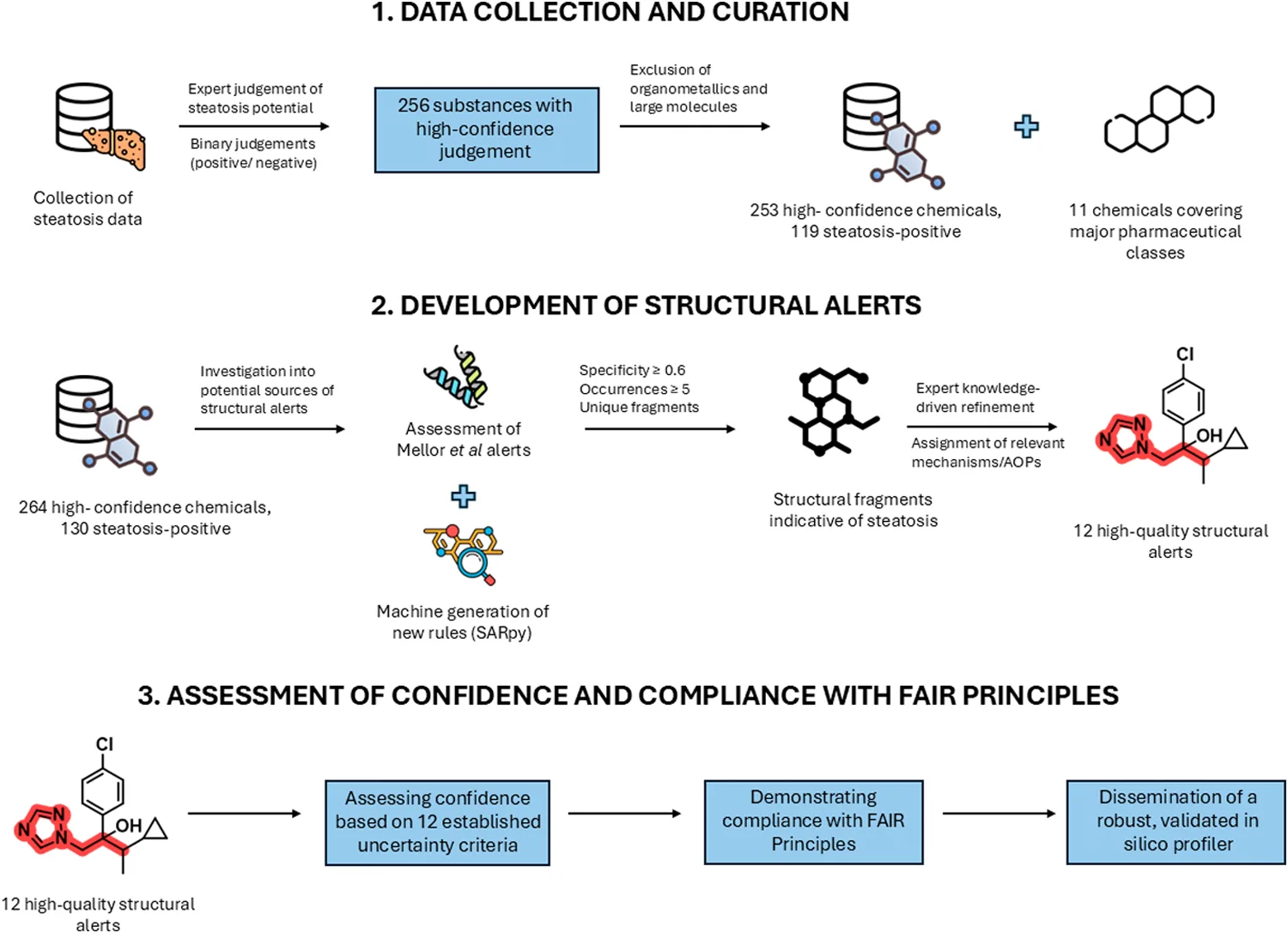
A schematic depiction of the workflow. Following curation, SARpy-generated fragments derived from 253 curated chemicals were, alongside the rules described in Mellor et al., investigated as potential sources of structural alerts. Manual refinement following application of predefined exclusion criteria yielded ten mechanistically grounded alerts, for which confidence and compliance with FAIR Lite principles were assessed. Two additional alerts covering previously underrepresented pharmaceutical classes were constructed later.

Although not intended as a predictive model, the 214 alerts presented in Mellor et al.^21^ were evaluated as such using the curated data set. The performance of these is presented in Table S4A of the Supporting Information. When assessed strictly as a predictive model, they demonstrated limited performance, with most unable to meet the criteria defined in Section 2.2. This observation necessitated the generation of additional fragments, using the curated data set as training data, for the purposes of supplementing and expanding upon them. Fragments identified through this process were refined using expert judgment, such that, where possible, each fragment captured a distinct grouping of structurally related chemicals. This led to the development of ten structural alerts that were further supplemented with two alerts constructed at a later stage, and these 12 alerts together constitute the *in silico* profiler. Described within Table 2 are each of these, accompanied by details relating to their chemistry, putative associated MIEs and AOPs, measures of performance, and usage profiles of the chemicals captured. An in-depth description of each alert follows, with further details relating to the matched substances provided in Table S5 of the Supporting Information.

**Table 2:** Alert Descriptions, Putative Mechanisms of Action, Relevant AOPs, Representation among Steatosis-Positive and Steatosis-Negative Compounds, and Typical Uses of the Chemicals Captured by the 12 Alerts^a^

Chemistry	Alert ID	Putative Mechanism of Action	Relevant AOP/s	TP	FP	Precision	Typical Uses of Captured Chemicals
Aryloxyphenoxypropionate derivatives	1	Activation of PPAR-α and PPAR-γ	529	4	0	1	Selective herbicides
N-substituted triazoles	2	PXR activation	60	19	1	0.95	Fungicides
Branched alkyl carboxylic acids	3	Modulation of PPAR signaling	529	6	0	1	Antiepileptic (valproic acid); solvents for industrial applications
Nucleoside analogues	4	Inhibition of mitochondrial DNA polymerase	-	6	1	0.86	Antivirals
Amide-linked aromatic and polar rings	5	PXR activation	60	4	0	1	Fungicides
Biaryl structures with a carbon linker	6	Interaction with ER, AR, PXR, and CAR	58, 60	7	0	1	Tamoxifen (anticancer), pesticides, and insecticides
Pyrethroids	7	Modulation of AMPK and FXR-PPAR-α −CPT1 pathways	61, 529	2	1	0.67	Insecticides
Cationic amphiphilic structures	8a	Dysregulations of AR, HNF4α, RXR, NR2F1, and PPAR-α	529	2	0	1	Tamoxifen (anticancer) and Amiodarone (antiarrhythmic)
	8b	Multiple mechanisms reported	34, 518, 529	2	0	1	Antipsychotics
Phenylureas and benzoylureas	9	PPAR-γ agonism	529	4	3	0.57	Herbicides and insect growth regulators
Oxadiazoles	10	Unclear at present	-	2	0	1	Oxadiazole herbicides
Corticosteroids	11	GR activation	318	7	0	1	Anti-inflammatory and immunosuppressive agents

a AMPK: AMP-Activated Protein Kinase, AR: Androgen receptor, CPT: Carnitine Palmitoyl Transferase, ER: Estrogen Receptor, FP: False Positive, GR: Glucocorticoid Receptor, HNF4α: hepatocyte nuclear factor 4α, LXR: Liver X Receptor, MIE: Molecular Initiating Event, NR2F1: Nuclear receptor subfamily 2 group F member 1, PPAR: Peroxisome Proliferator Activated Receptor, PXR: Pregnane X Receptor, RXR: Retinoic Acid Receptor, and TP: True Positive.

#### 3.2.1 Aryloxyphenoxypropionate Derivatives

Aryloxyphenoxypropionates find use within the agrochemical industry as selective herbicides. Typically composed of oxygen-linked aryl/heteroaryl rings, the ‘fop’ label is derived from the shared phenoxy unit.^41^ Although their herbicidal activity is mediated through the inhibition of acetyl-CoA carboxylase present within the plastids of target species, they have also been shown to increase expression of the nuclear receptors PPAR-α and PPAR-γ in primate CV-1 cell lines and further to induce adipogenesis, lipid accumulation, and prevention of lipid breakdown in murine 3T3-L1 adipocytes and within *in vivo* rodent models.^42,43^ Alert 1 (Figure 3) captures four members of this class: diclofop-methyl, fluazifop, propaquizafop, and quizalofop-P-tefuryl. All show a positive association with the emergence of steatosis and are present in the EFSA Steatosis CAG, with the lowest observable adverse effect levels (LOAELs) of ≤100 mg/kg/day.^44^

**Figure 3:**
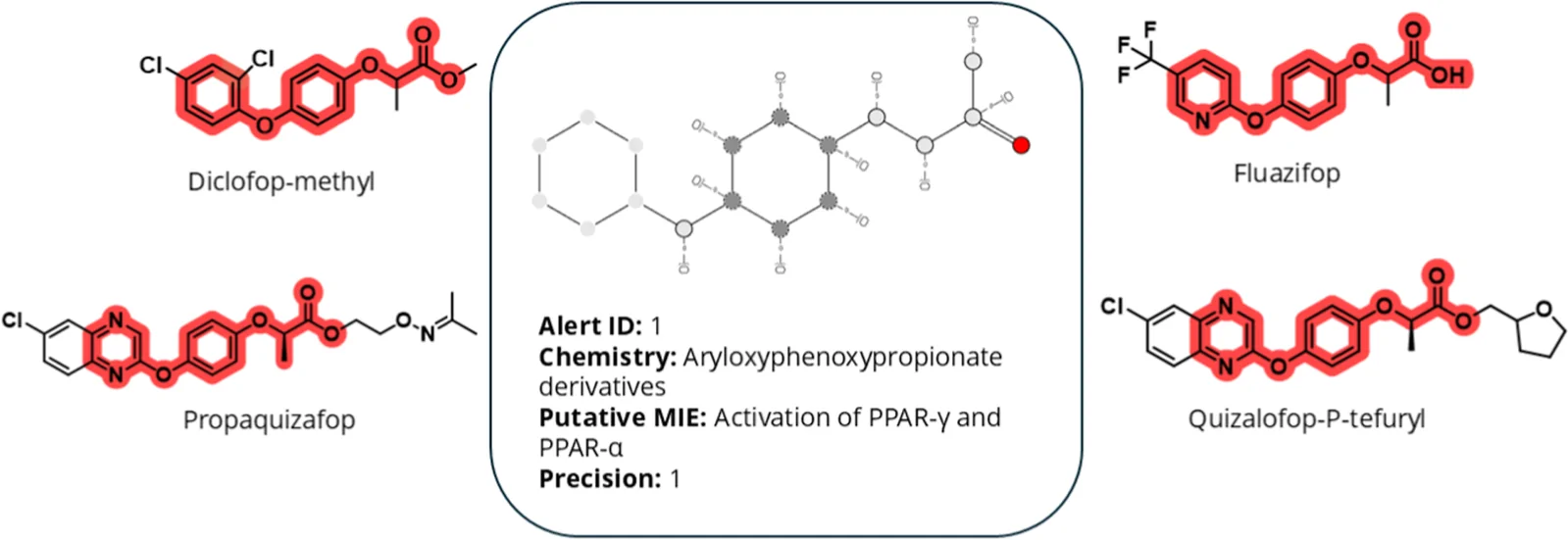
Structural alert 1 and its definition, alongside chemicals captured by the alert. Four aryloxypropionate analogues are captured, all of which are associated with steatosis.

#### 3.2.2 Azole Antifungals

Azole antifungals are used primarily within agriculture to protect plants from fungal infection. Exposure to these chemicals is associated with steatosis, with representatives such as propiconazole and tebuconazole mechanistically linked to PXR activation in human liver cell lines—potentially leading to triglyceride accumulation and then to steatosis.^45^ Alert 2 (Figure 4) defines an alkyl substituent on a five-membered aromatic nitrogen-containing ring, typical of imidazole, pyrazole, and triazole systems. It demonstrates strong performance, identifying 20 substances within the data set, 19 of which were judged to be associated with steatosis.

**Figure 4:**
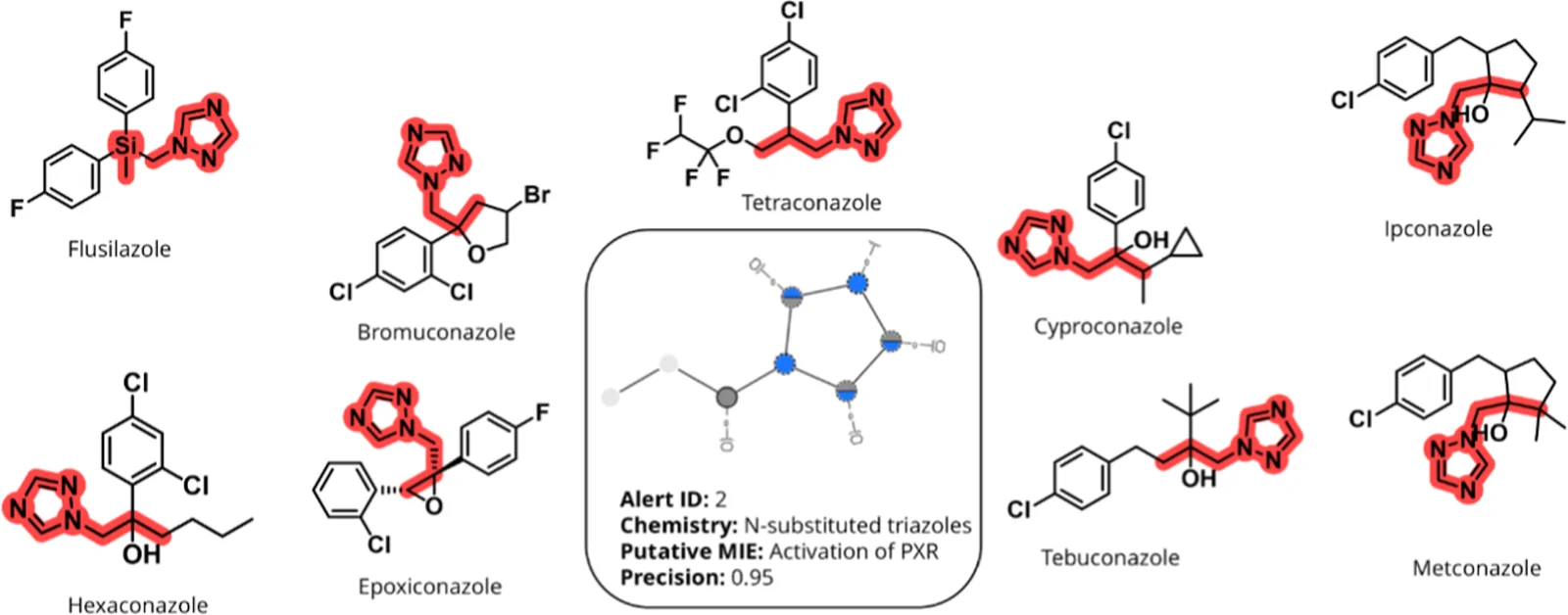
Alert 2 and its definition, together with chemicals captured.

#### 3.2.3 Branched Alkyl Carboxylic Acids

The antiepileptic drug valproic acid and its analogues are well-known inducers of hepatic steatosis. Various MIEs and mechanisms have been proposed, such as triglyceride accumulation arising through increased expression of Nrf2, the upregulation of PPAR-γ, and also C-036-dependent lipid uptake preceding lipid droplet formation.^46^ Alert 3 (Figure 5) describes branched aliphatic carboxylic acid analogues with side chains of varying lengths and correctly identifies six members of this class (including valproic acid) with no misclassifications. All of these compounds showed similar gene expression profiles in transcriptomics analysis, supporting the notion that they induce steatosis via modulation of fatty acid metabolism, PPAR signaling, and stress response.^28^

**Figure 5:**
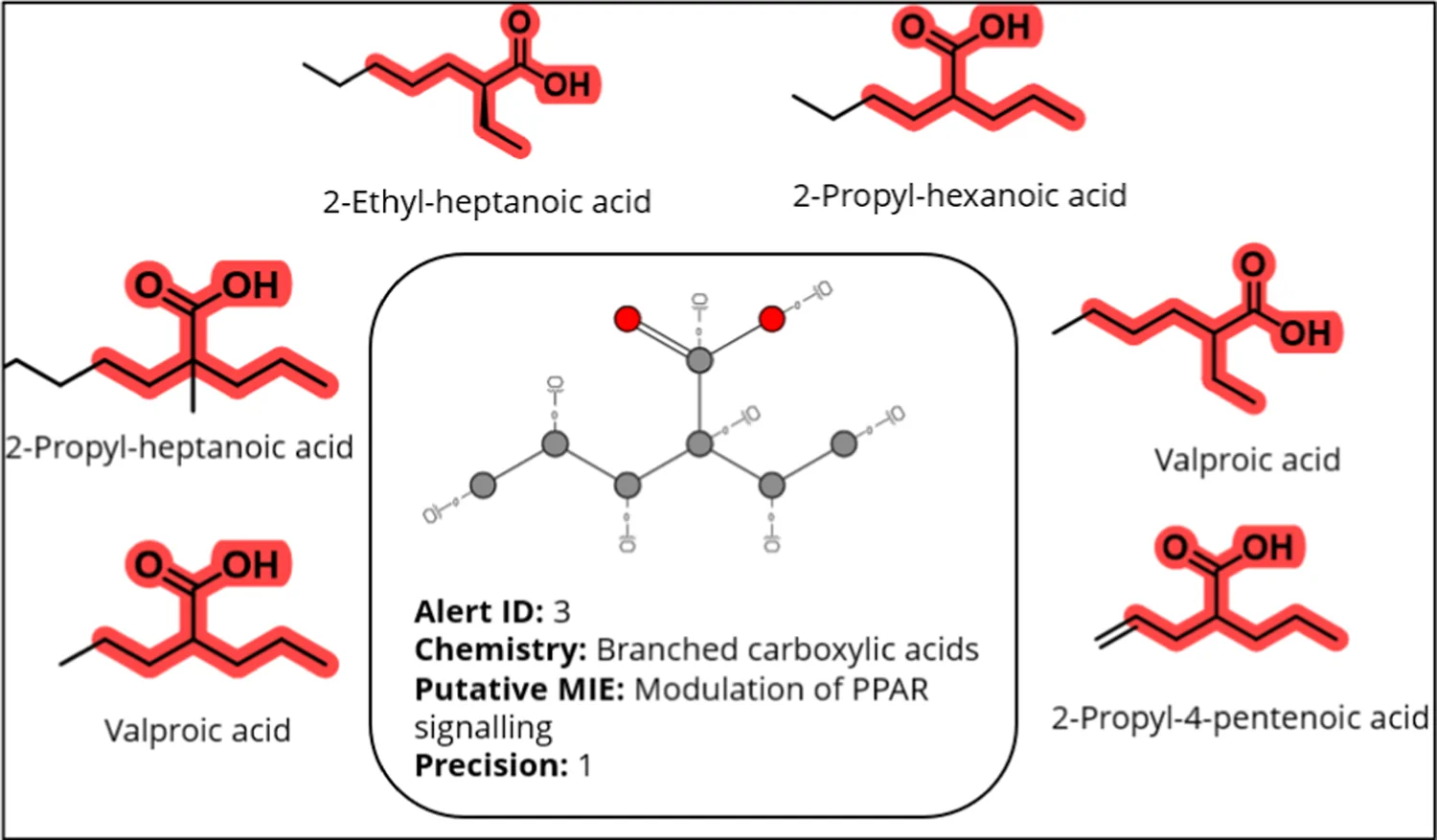
Alert 3 and its definition, alongside chemicals that trigger it.

#### 3.2.4 Nucleoside Analogues

Forming the building blocks of nucleic acids, nucleosides are composed of a five-membered cyclic sugar unit linked to a nitrogenous base (such as purine or pyrimidine). Alert 4 (Figure 6) describes a nucleoside-like structure, consisting of a furanose and deoxyribose sugar ring attached to an aromatic nitrogen, representative of those present in such bases. Seven compounds containing this motif were identified in the data set, six of which are associated with steatosis. Hepatotoxicity of these substances is well-documented, an exemplar case being the discontinuation of fialuridine owing to hepatotoxicity-related deaths in patients with chronic hepatitis B. These effects are thought to occur due to mitochondrial injury arising through inhibition of mitochondrial DNA polymerase, leading to acute micro- and macrovesicular steatosis, lactic acidosis, and related complications.^47^

**Figure 6:**
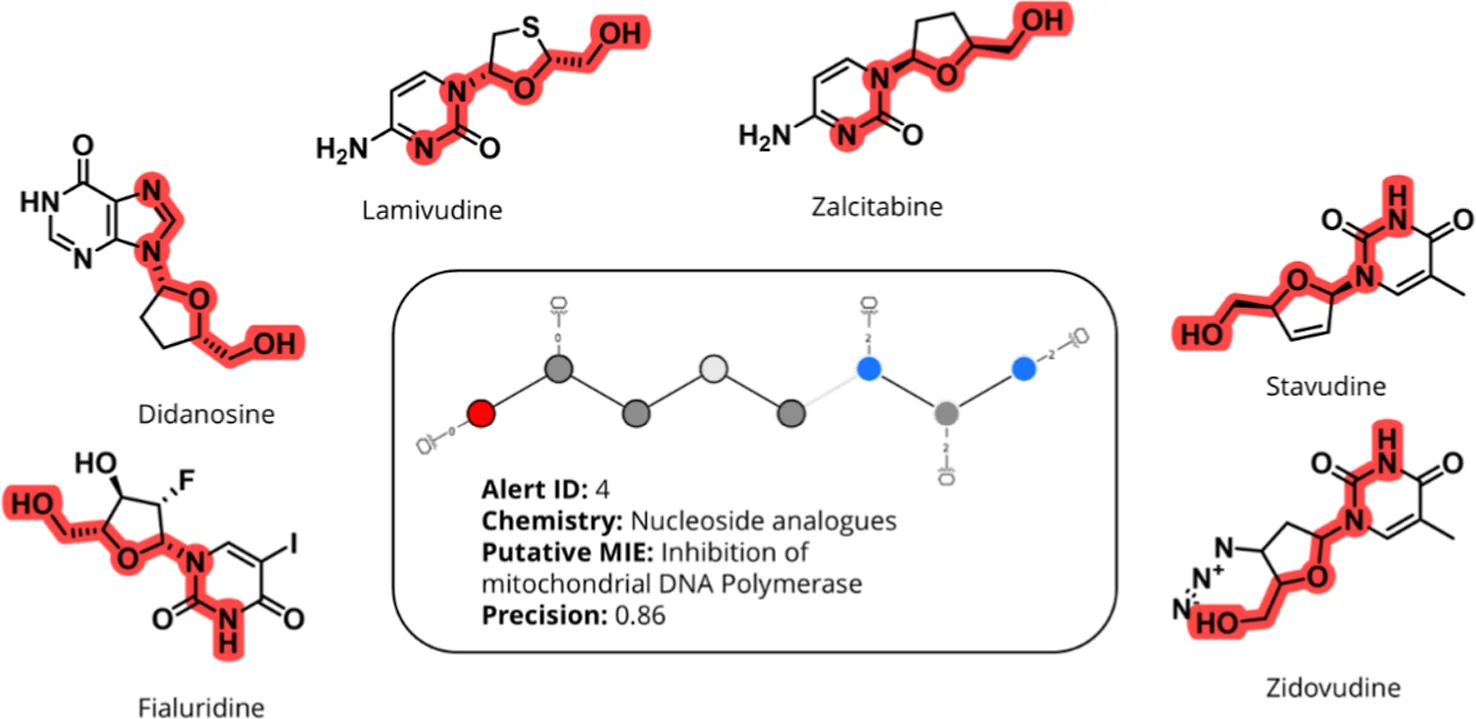
Alert 4 and its definition together with chemicals captured.

#### 3.2.5 Succinate Dehydrogenase Inhibitors (SDHIs)

Inhibitors of succinate dehydrogenase, an enzyme involved in glucose metabolism and ATP production within eukaryotes, are an important class of agrochemical fungicides.^48^ Such compounds are typically composed of a pyrazole ring and a further aromatic moiety, separated by an amide linkage.^49^ These features, contained within Alert 5 (Figure 7), capture three members of this class (bixafen, fluxapyrad, and isopyrazam), alongside the chloroacetamide herbicide metazachlor. There is strong evidence linking SDHIs to PXR activation, which seems the likely molecular mechanism by which they induce steatosis.^50^

**Figure 7:**
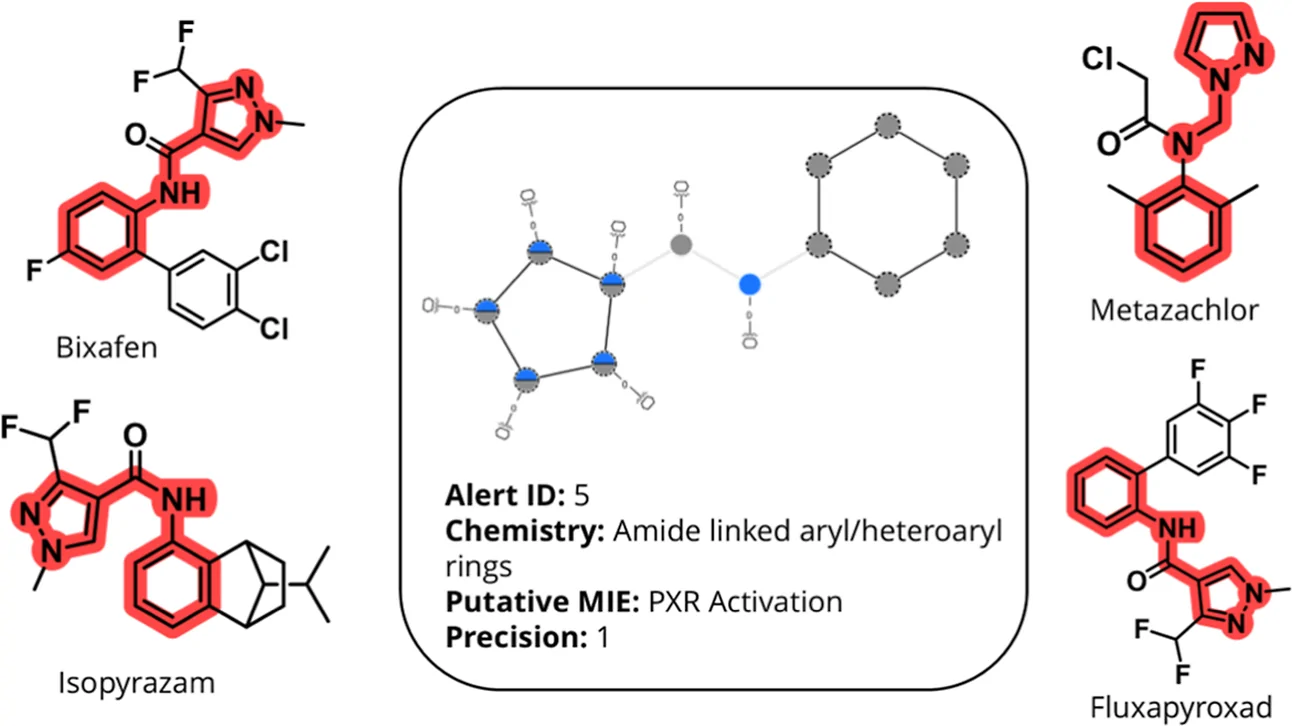
Alert 5 and its definition, together with chemicals captured.

#### 3.2.6 Alert 6

Alert 6 (Figure 8) describes two benzene rings linked by a single carbon. All seven chemicals that trigger this rule are associated with steatosis. These include the organochlorine insecticides dichlorodiphenyltrichloroethane (DDT) and dichlorodiphenyldichloroethylene (DDE), the anticancer drug tamoxifen, and some lesser-studied fungicides. Despite their ban in the 1970s, DDT and DDE continue to persist in the environment owing to their accumulative properties.^51^ Interactions with several nuclear receptors have been implicated in the development of steatosis associated with exposure to each, including the estrogen receptor, androgen receptor, pregnane X receptor, and the constitutive androstane receptor.^52–55^ Proposed mechanisms include dysregulation of genes involved in fatty acid uptake (CD36), lipid metabolism (ALB, AKT1), and fatty acid biosynthesis (Scd2 and TP53).^51,56^ Although the alert correctly identifies chemicals associated with steatosis, it is acknowledged that these do not share structural features beyond the biaryl motif captured within. As such, it does not define a specific chemical class. This limitation must be borne in mind when considering usage for purposes such as grouping or read-across.

**Figure 8:**
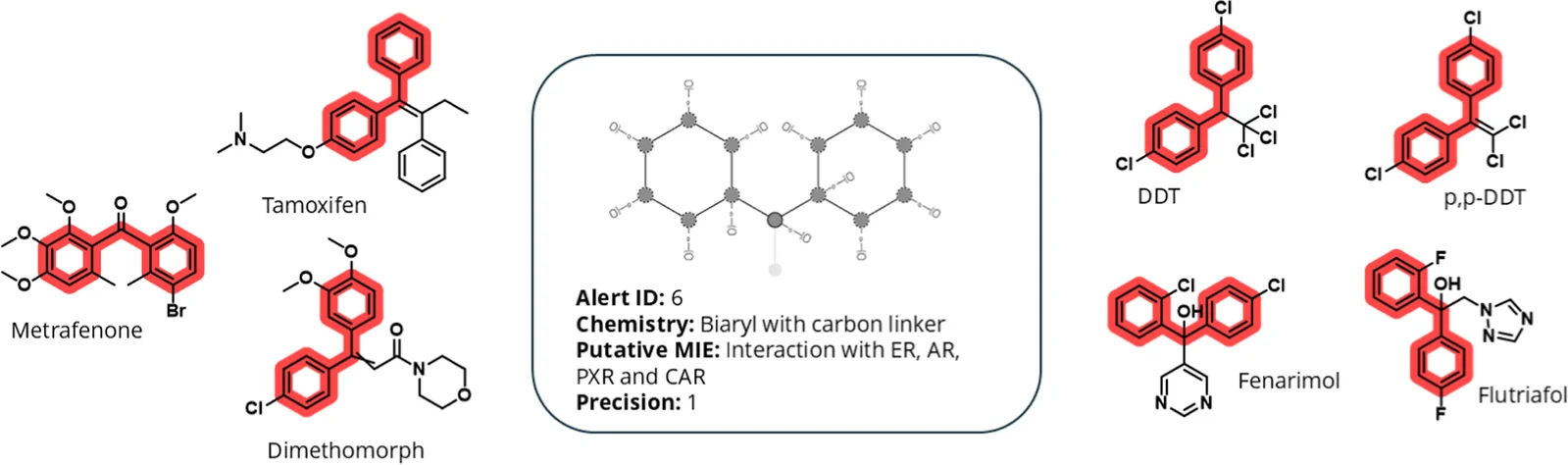
Alert 6, its definition, and chemicals captured.

#### 3.2.7 Pyrethroids

Alert 7 (shown in Figure 9) was designed to capture pyrethroid insecticides, which are synthetic analogues (typically cyclopropanecarboxylic acid derivatives) of naturally occurring pyrethrins found in *Chrysanthemum* flowers.^57^ The alert captures three substances (permethrin, cypermethrin, and deltamethrin) two of which associate positively with steatosis. Exposure to pyrethroids has been found to result in increased triglyceride accumulation and fatty acid synthesis, alongside dysregulated lipid metabolism in HepG2 cells. Impairment of other liver functions, such as glucose metabolism, is also noted—as is the induction of dyslipidaemia and the emergence of endoplasmic reticulum stress. Proposed mechanisms include AMP-activated protein kinase (AMPK)-dependent induction of lipogenesis and reduced fatty acid oxidation by downregulation of the FXR-PPAR-α-carnitine palmitoyl transferase-1 (CPT-1) pathway.^58^

**Figure 9:**
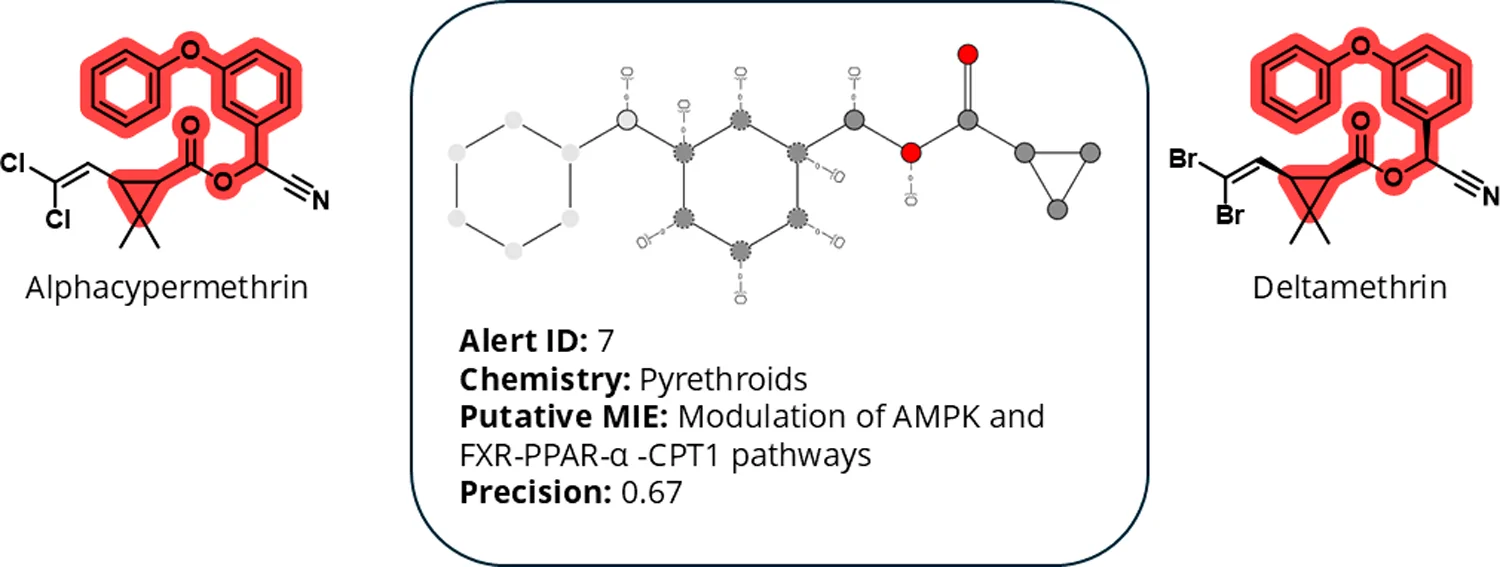
Alert 7 and its definition, alongside chemicals captured.

#### 3.2.8 Cationic Amphiphilic Drugs (CADs)

CADs are characterized by the presence of one or more lipophilic rings (LogP >1), alongside a secondary or tertiary amine (p*K*_a_> 6.5).^59^ These structural features promote ionization in the acidic environment of lysosomes, leading to inability to diffuse across intracellular membranes. This can result in their accumulation within the liver, thus predisposing to various forms of hepatotoxicity. Alert 8a (Figure 10) captures two CADs: the antiestrogen agent tamoxifen and the antiarrhythmic amiodarone. Alongside their parallel association with phospholipidosis, CADs are strongly linked to steatohepatitis, an advanced form of steatosis characterized by both excessive lipid build-up and inflammation.^60^ *In vivo* studies have demonstrated increased synthesis of fatty acids and marked triacylglycerol accumulation in rodents following exposure to tamoxifen, with gene expression analysis linking steatosis to dysregulation of the androgen receptor, the hepatocyte nuclear factor 4α (HNF4α), the retinoic acid receptor, and the nuclear receptor subfamily 2 group F member 1 (NR2F1).^61,62^ Amiodarone, used widely in the treatment of atrial fibrillation, is thought to induce steatosis via several mechanisms. These include decreased fatty oxidation via inhibition of CPT-1-mediated transport of long-chain fatty acids across mitochondrial membranes, and decreased lipoprotein export via inhibition of mitochondrial triglyceride transfer protein.^60^ Analysis of hepatic RNA in amiodarone-treated mice showed significant microvesicular lipid accumulation, correlated with increased expression of genes regulated by PPAR-α.^63^

**Figure 10:**
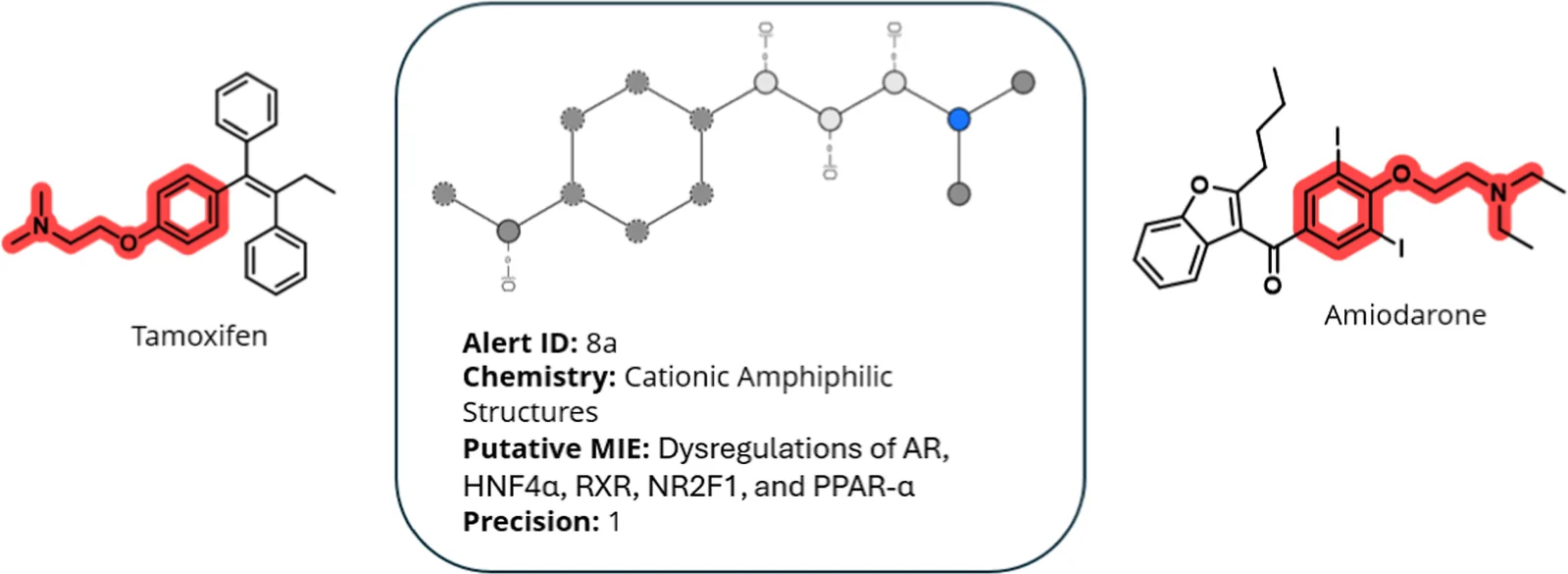
Alert 8a and its definition, alongside the two chemicals captured.

Alert 8b (Figure 11) was constructed to capture the atypical antipsychotics clozapine and olanzapine, which are thought to promote steatosis through multiple interrelated mechanisms. Exposure to clozapine *in vivo* has been found to cause marked dysregulations of PPAR, LXR, and sterol regulatory element binding protein-1­(SREBP-1) controlled gene expression.^64^ At the hepatic level, clozapine has been reported to elevate interleukin-1 and C-reactive protein levels and to inhibit adipogenesis, promoting synthesis of very low density lipoproteins (VLDLs) and TGs.^65^ Similarly, olanzapine exposure is known to result in TG accumulation and inhibition of mitochondrial β-oxidation, both characteristic features of hepatic steatosis. Posited mechanisms include activation of SREBP-1 and 2, downregulation of apolipoproteins A and B, inhibition of CPT1A, and upregulation of sortilin1 (SORT1), among others.^65^ A more detailed exploration into the mechanisms of steatosis induction by atypical antipsychotics may be found in Gonzalez et al.^65^

**Figure 11:**
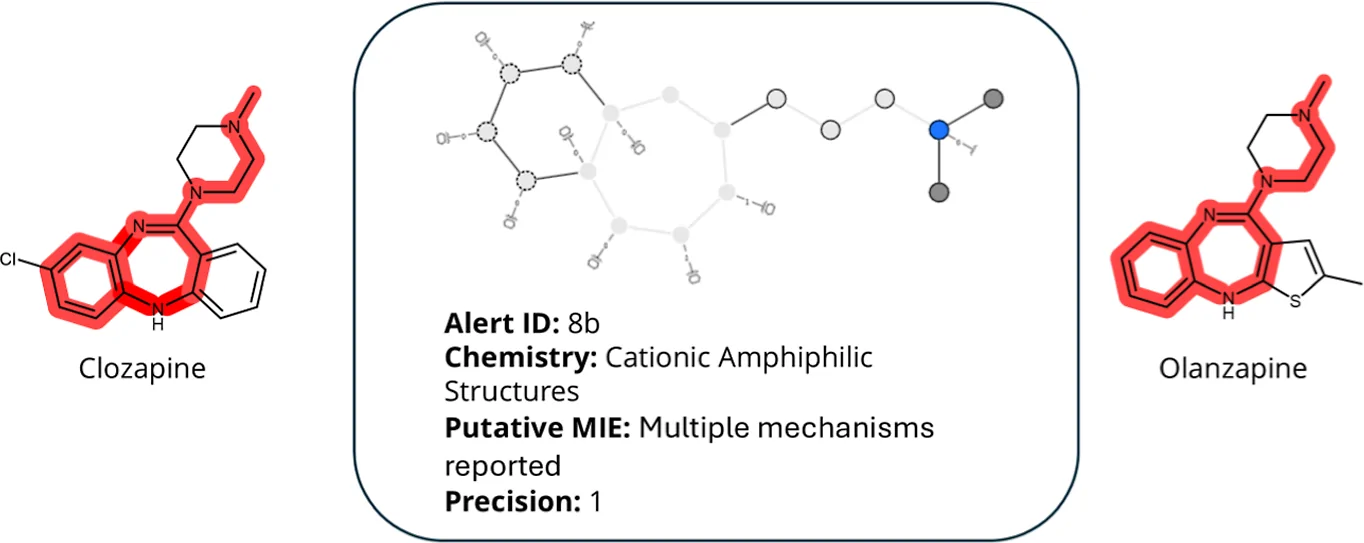
Alert 8b and its definition, alongside chemicals captured.

#### 3.2.9 Phenylureas and Benzoylureas

Phenylureas and N-benzoyl-N’-phenylureas (BPUs) are important classes of agrochemicals, with the latter, in particular, used widely in crop protection as insect growth regulators owing to their non-neurotoxic mode of action (this being the inhibition of chitin biosynthesis).^66^ Evidence has emerged linking BPUs such as flufenoxuron and diflubenzuron to potent agonism of PPAR-γ in HepG2 cell lines, leading to decreased expression of genes encoding the tricarboxylic acid cycle-participating enzymes citrate synthase, oxoglutarate dehydrogenase, and pyruvate dehydrogenase alpha 1. These changes are thought to increase availability of glycerol precursors, promoting triglyceride accumulation and, eventually, steatosis.^42^ Alert 9 (Figure 12) was constructed to capture the characteristic phenylurea subunit common to these classes, with the occurrence of steatosis associated with four of the seven substances bearing the group.

**Figure 12:**
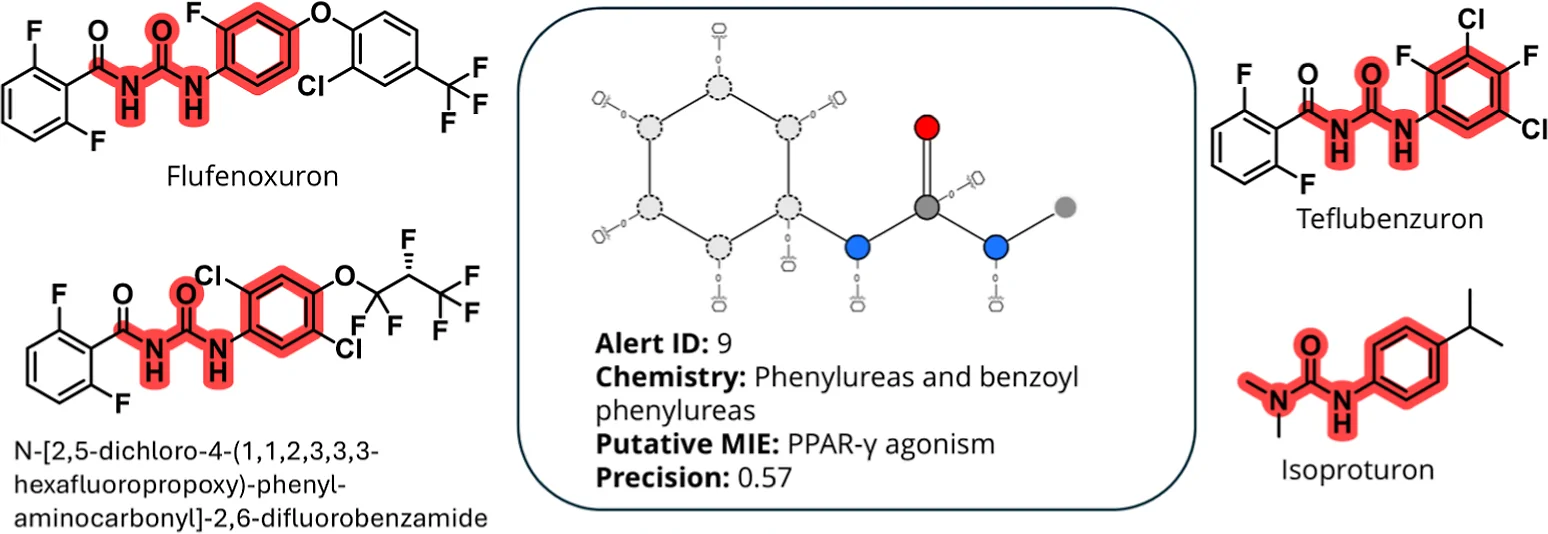
Alert 9 and its definition, alongside chemicals captured.

#### 3.2.10 Oxadiazoles

Alert 10 relates broadly to a structural motif consisting of a six-membered aromatic ring further linked to a five-membered ring (Figure 13) by means of a single bond. It captures two oxadiazoles (oxadiargyl and oxadiazon), each of which is steatosis-positive and finds use as broad-spectrum herbicides through their inhibition of protoporphyrinogen oxidase.^67^ While oxadiargyl has been classified by EFSA into CAG2b, indicating a propensity to cause hepatocellular fatty changes *in vivo*,^34^ little is known concerning the mechanisms by which these chemicals induce steatosis. Further evidence is needed to allow conclusions about the hepatotoxic effects of this group.

**Figure 13:**
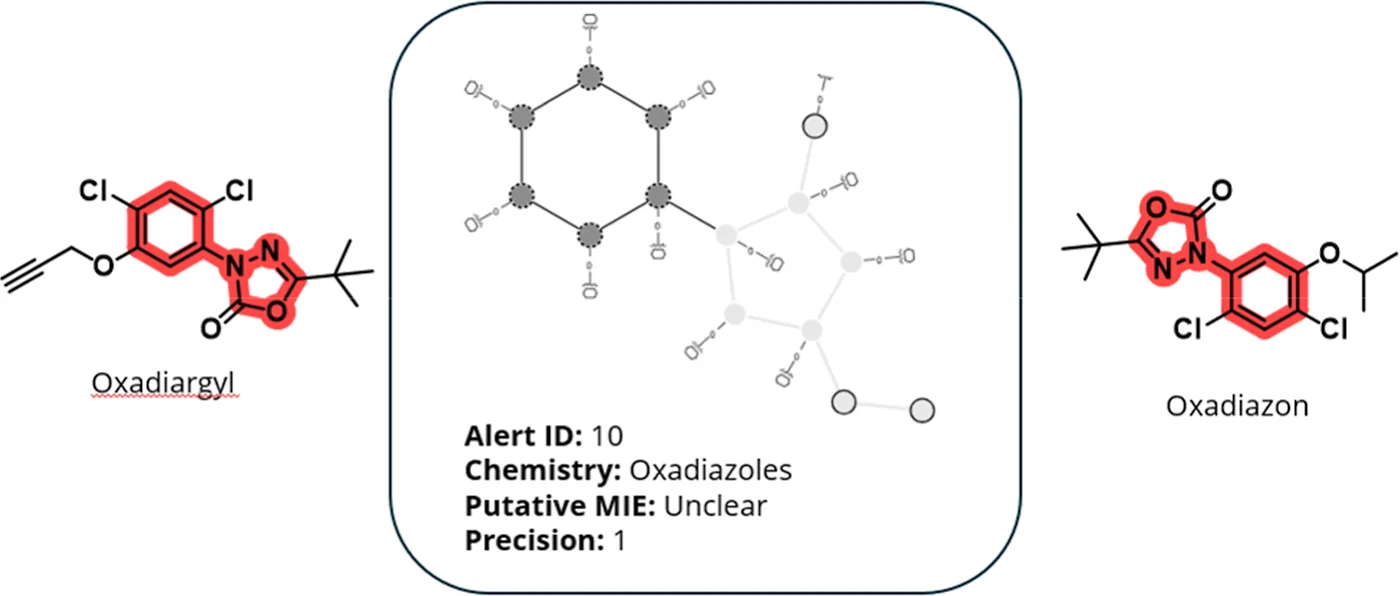
Alert 10 and its definition, alongside chemicals captured.

#### 3.2.11 Corticosteroids

Corticosteroids are a class of natural and semisynthetic steroids widely used in medicine on account of their potent immunosuppressive and anti-inflammatory properties.^68^ Seven corticosteroids, holding positive associations with steatosis induction, were retrieved from the literature (listed in Table S2 in the Supporting Information). These were captured through construction of an alert bearing the pregnane ring characteristic of these compounds (Figure 14). Corticosteroid therapy is known to promote steatosis, alongside other hepatic complications, particularly when prescribed long-term and at higher doses.^68^ Induction of steatosis is thought to proceed largely via activation of the glucocorticoid receptor,^69^ leading to decreased mitochondrial fatty acid beta oxidation, triglyceride accumulation, and steatosis (as is described in AOP 318).^70^

**Figure 14:**
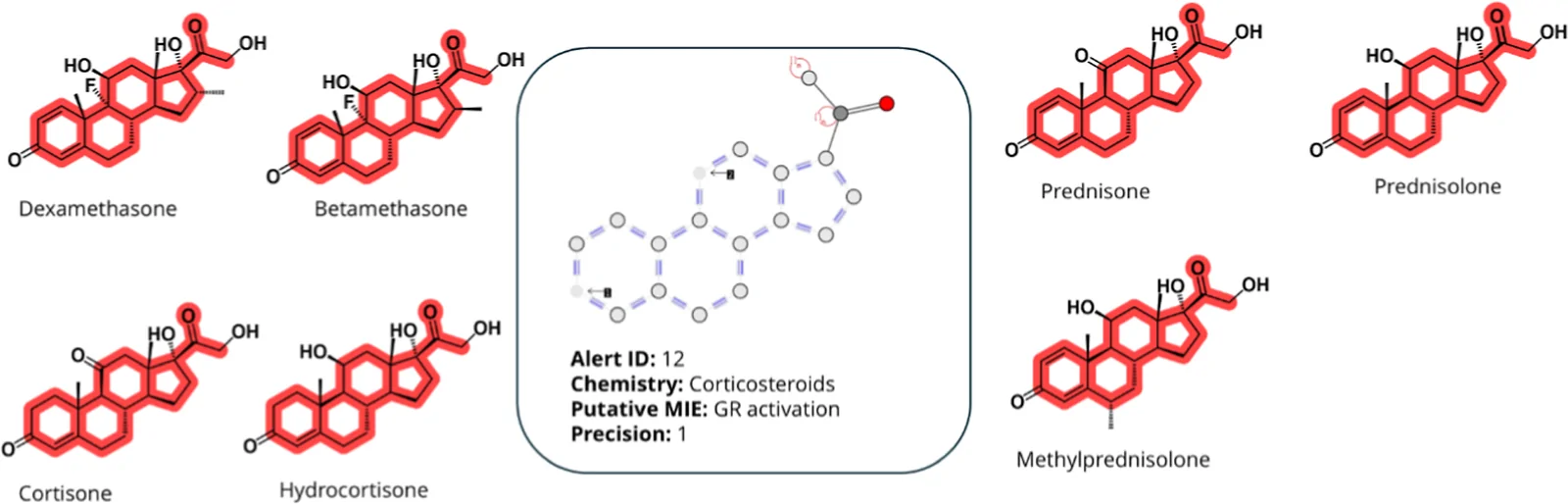
Alert 11 and its definition, alongside chemicals captured.

### 3.3 Assessment of Confidence within the Structural Alerts

An overall assessment of the profiler, in terms of confidence held in relation to aspects of uncertainty, is presented in Table 3. Individual analyses for each alert are further presented in Table S6 of the Supporting Information. It is important to bear in mind the intended purpose of the structural alerts when assessing accompanying uncertainty, and different levels of confidence may be considered acceptable based upon the use case. Cronin et al.^23^ propose five essential attributes of a structural alert intended for hazard identification and risk assessment, which must, ideally, be held with high confidence. It is noteworthy that the alerts presented in this study are associated with high confidence in all these areas (structural description, property domain, toxicity or relationship to adversity, species specificity, and corroborating evidence). It is acknowledged that low confidence is apparent in associated metabolic understanding. However, given the intended use-case, this may be considered acceptable and, in the authors’ opinion, should not preclude their adoption.

**Table 3:** Assessment of the Overall Level of Confidence of the Structural Alerts Using the Criteria Described in Cronin et al.^23^ and Associated Comments and/or Improvement Strategies^a^

Criteria	Overall Confidence Level and Reasoning	Comments and/or Improvement Strategies
Toxicity or Relationship to Adversity	High: alerts relate to a clearly defined adverse effect	Intended to support risk assessment for hepatic steatosis, a well-defined adverse outcome characterized by excess lipid accumulation within the liver
Purpose	High: alerts have a clearly stated purpose outlining correct usage	Intended to support risk assessment through hazard identification (e.g., weight of evidence in a NAM-based tiered testing strategy), mechanism-based analogue identification, and guide read-across
Structural description	High-the chemistry of the alerts is unambiguously described	The chemistry of the alerts and their local molecular environments (e.g., ring substitution patterns, branching, etc.) is described clearly
Property Domain	High: alerts have a clearly defined physicochemical-property domain	Domain of the alerts defined in terms of six physicochemical properties derived from training data: XLogP, molecular weight, HBA, HBD, nRotB, and TPSA (Tables S3A and S3 B)
Species Specificity	High-the species to which the toxicity is relevant has been stated clearly	Alerts associated with toxicity in rodents and may be extrapolated to humans through appropriate scaling approaches
Metabolic Domain	Low-metabolic considerations not explicitly incorporated	Unclear whether metabolic activation is required for toxicity, in part due to the multitude of mechanisms that may contribute to the development of steatosis
Mechanistic Interpretation	High: alerts are associated with clearly definable mechanisms of toxic action	Alerts supported with a clear mechanistic rationale through an extensive literature search and, where possible, AOPs for steatosis (alert 10 being an exception)
Mechanistic Causality	Moderate to high-chemistry captured by alerts generally relate to mechanisms of toxic action	Structural chemistry of alerts generally relates to MIEs of the mechanisms/AOPs for steatosis (except alerts 8a and 10)
Coverage	High: alerts expected to operate within a well-defined domain	Alerts refined using expert judgment to ensure optimal balance between specificity and generality of coverage
Performance	High-the alerts have good performance	Alerts have high precision and low false positive rates (precision ≥0.8 for 10 of 12 alerts)
Corroborating Evidence	High-multiple toxicological data support the alerts	Confirmatory toxicological data from regulatory databases (RepDose, ToxRefDB), EFSA CAG2b classifications, and *in vitro* and *in vivo* studies
Supporting Evidence	High: alerts supported with strong mechanistic evidence	Alerts supported with references to *in vitro* data and *in vivo* assays confirming mechanisms, e.g., nuclear receptor activation, mitochondrial dysfunction (except alerts 8 (limited evidence) and 10)

a NAM: new approach methodology, HBA: H-bond acceptors, HBD: H-bond donors, XLogP: logarithm of octanol/water partition coefficient, nRotB: number of rotatable bonds, and TPSA: topological polar surface area.

### 3.4 Demonstrating Compliance with FAIR Lite Principles

Presented within Table 4 are the FAIR Lite principles, as outlined in Cronin et al.,^25^ alongside the measures taken to ensure that the alerts developed in this study satisfied these criteria. To assist in “Findability”, the alerts were assigned a globally unique digital object identifier. Details in relation to capture and curation, including applicability domain and performance estimates, have been clearly documented to enable reusability and interoperability. To ensure transparency, metadata, both dependent (sources from where the chemicals and associated toxicity data were derived) and independent (structural fragments prior to refinement), have been provided in Tables S2 and S4, respectively. To comply with the final FAIR Lite criterion, alerts were stored in a repository, allowing for their ready retrieval.

**Table 4:** Assessment of the Alerts with Regard to Compliance with the FAIR Lite Principles

FAIR lite principle	Checklist	Steps to Ensure Compliance
Unique Identifier	Provision of a unique identifier	Profiler assigned a globally unique and persistent digital object identifier (10.5281/zenodo.20236678)
Model Capture and Curation	Description and provision of the model/s in a standardized format	Detailed descriptions of the model provided along with recommendations for correct usage
	A clearly stated applicability domain	Model applicability domains defined in terms of physicochemical properties derived from training data
	Provision of model performance data	Performance metrics (true positives, false positives, and precision) stated clearly
Metadata	Provision of model-associated dependent data	Toxicity data described, along with links/references to sources from where these data were retrieved
	Provision of model-associated independent data	Structural features identified in the original Mellor paper, along with fragments generated by SARpy, are each provided
	Use of standardized ontologies to describe metadata	Standardized ontologies used wherever possible to describe dependent and independent data
	Licensing details (if any)	Appropriate licensing options (Creative Commons) selected to facilitate use
Storage	Storage of information pertaining to the model, data, and license	All information related stored and freely accessible upon the Zenodo repository and (10.5281/zenodo.20236678)
	Availability of codes/workflows related to the model	KNIME workflow used in evaluation of alerts not publicly released at this stage (intended as separate publication). Alerts described in full, with SMARTS patterns, associated data, and methodological details provided to enable independent implementation
	Integration with other software to enable usage	SMARTS pattern for alerts provided, may be integrated into commonly used cheminformatics platforms (e.g., KNIME)

### 3.5 Recommendations for Practical Use

To support correct application of the profiler, the following recommendations are made. These build upon the intended purpose of the alerts and current use patterns.

a. Interpreting an Alert Hit

An alert hit may be considered to convey the following.

- A preliminary binary indication of steatosis potential. It must be noted that this is not a quantitative prediction and should not be interpreted as such.
- Mechanistic insights, in that a chemical that triggers an alert may be linked to MIEs and, where established, associated AOPs for steatosis. However, chemicals are not restricted to a single mode of action, and an alert hit should not exclude the involvement of additional mechanisms.
- A basis for grouping, read-across, and mechanism-based analogue identification. This is of particular relevance to Alerts 9 and 10.

b. Interpreting a “no hit”: Absence of an alert hit should not be taken to indicate that a chemical is not steatotic. Negative alerts were not constructed as part of this work, and a proportion of steatosis-positive chemicals within the data set are currently not covered by the profiler. A “no hit” outcome should therefore be considered as inconclusive, and additional evidence should be sought to inform decision-making.

c. Weight of evidence: While the profiler may be useful as a standalone tool, it is recommended that the profiler be integrated within a weight of evidence approach alongside complementary *in silico* and *in vitro* models as part of a broader NAM-based tiered testing strategy. Different strategies may be required for molecules where alerts are hit, *i.e.*, to confirm a mechanism of action, or are absent, *i.e.*, to confirm no specific toxicity.

d. Alert specific considerations: Performance of individual alerts varies in terms of precision, number of chemicals captured, and species to which the underlying toxicological data are relevant (Table S2). These must be reviewed prior to their use.

e. Applicability domain: It must be verified that query chemicals fall within the chemical property domain of the profiler (Table S3B). Use of the profiler is not precluded for chemicals outside its domain; however, outcomes generated shall be associated with lower confidence.

f. Limitations:

It is acknowledged that the profiler in its present form has limitations. These are summarized below and must be borne in mind by the user.

- The relatively small size of the data set precluded external validation, usually performed by holding out 20% of the data set. As such, performance estimates are perhaps optimistic, given that the alerts were both developed and evaluated on the same set of chemicals. It is intended that the profiler be more rigorously validated using an independent test set as more steatosis data become available.
- The coverage of the profiler is relatively restricted, and only a proportion of the steatosis-positive chemicals within the data set are currently captured. This is in part due to the exclusion criteria applied during alert development and the observation that steatosis occurs via a multitude of mechanisms; and it is challenging to cover all these mechanisms within a single profiler. To partially address the issue of coverage, the data set was expanded to improve coverage of major pharmaceutical classes associated with steatosis, which led to the development of two additional alerts (8b and 11). It is intended that the profiler be further expanded as more data become available.
- The alerts are assumed to be directly acting and presently do not account for metabolic activation. If metabolites of a query chemical are known or anticipated, then it is recommended that the profiler also be applied to these.

## 4 Discussion

The successful application of NAMs in next-generation risk assessment (NGRA) relies on the integration of information from different sources within a structured framework.^7^ As such, there is renewed focus on the development of test batteries that combine *in silico* predictions with *in vitro* tests as a means of yielding information concerning a chemical’s interactions with biological targets and pathways potentially leading to adverse outcome. Such frameworks, exemplified by the OECD Integrated Approaches to Testing and Assessment (IATAs)^71^ and the Alternative Safety Profiling Algorithm (ASPA),^72^ would increase confidence in the use of NAMs for regulatory risk assessment.

Against this backdrop, the present study sought to assess and improve upon a set of 214 MIE-derived rules indicative of hepatic steatosis, as developed by Mellor et al.^21^ An initial assessment revealed that most of these rules lacked selectivity, capturing common substructures and functional groups. It was therefore necessary to supplement the potentially more useful Mellor rules with SARpy-generated fragments derived from the curated chemical data set.

While several algorithmic approaches have been developed to generate substructure fragments indicative of toxicity,^73^ expert interpretation remains essential to ensure their mechanistic relevance.^74^ Therefore, the identified fragments were subsequently refined based on expert judgment. Such refinements are essential to enable extrapolation beyond the training data, thus ensuring that the broader context within which the defining fragments appear is appropriately captured. An example may be found within alert 2 (Figure 4), where requirements relating to atom type (presence of nitrogen/carbon at specified positions within the five-membered ring), aromaticity (ring atoms), and connectivity (restrictions on ring/total connections) are noted. Although challenging, such refinements are particularly important for end points such as hepatic steatosis, where noncovalent interactions with nuclear receptors, both electrostatic (e.g., H-bonding sites) and steric (e.g., bulk, shape, and flexibility), are linked to apical adverse outcome and must ideally be accounted for. The refinements made thus served to ensure that features liable to negatively influence such aspects of receptor binding are excluded, thereby increasing the mechanistic relevance and confidence in the predicted outcomes.

This approach, viz., the expert refinement of algorithmically generated fragments, led initially to the development of ten robust structural alerts. These were supplemented further with two alerts representing corticosteroid and tricyclic atypical antipsychotic drug classes. Thus, a total of 12 alerts comprised the profiler, for which a strong mechanistic underpinning was established through rationalization at the level of the MIE, consistent with the efforts to link chemistry to organ-level effects through MIEs and AOPs.^75,76^ These alerts do not replace the Mellor set but rather represent its refinement and extension, incorporating new fragments derived from the curated data set. It is thus intended that the outcomes associated with it shall, in addition to flagging potential hazard, offer insights into the mechanisms by which steatosis may develop. Furthermore, a comprehensive characterization of the uncertainties associated with each alert enhances confidence in overall suitability. Given their intended use-cases, i.e., hazard identification to support risk assessment and mechanism-based analogue identification, the profiler may be used with confidence. Moreover, compliance with the FAIR Lite principles ensures transparency and accessibility. From a methodological standpoint, this is, to the best of our knowledge, the first profiler to be disseminated for which confidence relating to aspects of uncertainty and compliance with FAIR Lite principles are formally assessed.

There are a number of potential use scenarios for the alerts, as identified in Section S5. Correct application is vital, and the set of alerts can provide a platform from which to start a weight of evidence, rather than being directly predictive. For instance, it is envisaged that the profiler presented should be used within tiered frameworks that integrate data from different *in silico* models, as opposed to being adopted as a standalone tool for direct assessment. Combining the mechanistic insights from the structural alerts within a weight of evidence approach that includes predictions from other *in silico* models, e.g., QSARs, molecular docking simulations, and quantitative *in vitro* to *in vivo* extrapolations (QIVIVEs), would guide further *in vitro* tests of relevance to characterizing steatosis progression. This has the potential to provide direct linkage of molecular features relating to MIEs to directed NAMs, for instance, within a tiered framework such as the ASPA.^72^ As noted in Section 3.5, different approaches may be required when an alert is hit, i.e., confirmatory, as opposed to the absence of an alert. Absence of an alert may require further information, within the context of a tiered strategy, in order to ensure that no toxicity has been missed.^77,78^ Alternatively, absence may provide a useful line of evidence when overall weight of evidence is considered.^10^

It is evident that the alerts relate to distinct groupings of chemicals, and the profiler may find use in identifying analogues for grouping and read-across.^79,80^ Furthermore, the defined applicability domain—derived from the physicochemical property ranges of the training data—affords a transparent means for evaluating whether a query chemical falls within the domain of the profiler. Defining the applicability domain of structural alerts has been attempted but is difficult without a defined data set upon which to base it.^81,82^

In summary, a robust, validated *in silico* profiler for hepatic steatosis is presented. Data for chemicals and their associations with steatosis were compiled, and through initial implementation of a computational workflow, followed by rigorous expert knowledge-driven refinement, these were employed in the development of ten robust structural alerts indicative of steatotic potential, expanded to include two additional alerts at a later stage. The resulting rules were supported with strong mechanistic evidence, linking them to MIEs and AOPs associated with the end point. Further characterization included an assessment of confidence, accompanied by a demonstration of compliance with the FAIR Lite principles recommended for computational toxicology models. It is hoped that the profiler will find use in toxicological risk assessment, particularly for hazard identification, analogue grouping, and read-across, and within weight-of-evidence approaches in tiered testing strategies. To enable usage, a web-based implementation of the steatosis profiler that requires only chemical structures as input is available at https://steatosis-profiler.streamlit.app.

## Supplementary Material



## Notes

Details of the data collection efforts, including chemical names, identifiers, and assignment of steatosis potential for 1378 substances; names, identifiers, and steatosis-related labels for chemicals within the data set used for generation and validation of structural rules; physicochemical properties for the chemicals within the data set; chemical property domain of the profiler; structural SMARTS patterns and associated nuclear receptors retrieved from Mellor et al.; SMILES strings for fragments generated using SARpy; descriptions, putative mechanisms of action, relevant AOPs, representation among steatosis-positive and steatosis-negative compounds, and usage of the 12 alerts post refinement; and assessment of confidence for each structural alert across areas of uncertainty using the 12 criteria described in Cronin et al. (XLSX)

## Notes

**Disclaimer** The opinions expressed in this document reflect only the authors’ views. The European Commission is not responsible for any use that may be made of the information it contains.

## Abbreviations

AhR: aryl hydrocarbon receptor
AOPs: adverse outcome pathways
AMPK: AMP-activated protein kinase
AR: androgen receptor
BPUs: N-benzoyl-N-phenylureas
CAG: cumulative assessment group
CAR: constitutive androstane receptor
CEBS: chemical effects in biological systems
CPT: carnitine palmitoyl transferase
DDT: dichlorodiphenyltrichloroethane
DDE: dichlorodiphenyldichloroethylene
EFSA: european food safety authority
ER: estrogen receptor
FAIR: findable, accessible, interoperable, and reusable
FP: false positive
FXR: farnesoid X receptor
GLP: good lab practice
GR: glucocorticoid receptor
HESS DB: hazard evaluation support system database
HNF4α: hepatocyte nuclear factor 4α
IATA: integrated approaches for testing and assessment (IATAs)
KE: key event
KNIME: konstanz information miner
LOAEL: lowest observable adverse effect level
LXR: liver X Receptor
MIE: molecular initiating event
NAMs: new approach methodologies
NGRA: next-generation risk assessment
NO­(A)­EL: no observable (adverse) effect levels
OECD: organisation of economic cooperation and development
PPAR: peroxisome proliferator activated receptor
PXR: pregnane X receptor
QIVIVE: quantitative *in vitro* to *in vivo* extrapolations
QSARs: quantitative structure–activity relationships (QSARs)
RXR: retinoid X receptor
ToxRefDb: toxicity reference database
TP: true positive
